# Review of the Nutrition Situation in Jordan: Trends and Way Forward

**DOI:** 10.3390/nu14010135

**Published:** 2021-12-28

**Authors:** Narmeen Jamal Al-Awwad, Jennifer Ayoub, Rawhieh Barham, Wafaa Sarhan, Murad Al-Holy, Mahmoud Abughoush, Huda Al-Hourani, Amin Olaimat, Ayoub Al-Jawaldeh

**Affiliations:** 1 Department of Clinical Nutrition and Dietetics, Faculty of Applied Medical Sciences, The Hashemite University, Zarqa 13133, Jordan; narmeen@hu.edu.jo (N.J.A.-A.); murad@hu.edu.jo (M.A.-H.); abulaith@hu.edu.jo (M.A.); hhourani@hu.edu.jo (H.A.-H.); aminolaimat@hu.edu.jo (A.O.); 2 Department of Nutrition and Food Sciences, Faculty of Agricultural and Food Sciences, American University of Beirut, Beirut 1107 2020, Lebanon; ja88@aub.edu.lb; 3 Nutrition Department, Ministry of Health, Amman 11118, Jordan; majeda_barham@hotmail.com; 4 Department of Nutrition and Food Technology, Faculty of Agriculture, Jordan University of Science and Technology, Irbid 22110, Jordan; wbsarhan17@agr.just.edu.jo; 5 Science of Nutrition and Dietetics Program, College of Pharmacy, Al Ain University, Abu Dhabi 64141, United Arab Emirates; 6 Regional Office for the Eastern Mediterranean, World Health Organization, Cairo 7608, Egypt

**Keywords:** nutritional status, malnutrition, infant and young child feeding, stunting, wasting, low birthweight, micronutrients, obesity, food consumption patterns

## Abstract

Jordan is witnessing an escalating pace of nutrition transition, which may be associated with an increased burden of malnutrition and related non-communicable diseases. This review analyzes the nutrition situation in Jordan by exploring specific nutrition indicators, namely infant and young child feeding, low birthweight, micronutrient deficiencies, anthropometric indicators, and food consumption patterns. Results showed that although most children were ever breastfed and early initiation of breastfeeding had a two-fold increasing trend, rates of exclusive breastfeeding below 6 months of age and continued breastfeeding until two years of age were low. Complementary feeding indicators, particularly minimum diet diversity and minimum acceptable diet standards, were suboptimal. An overall low burden of stunting, wasting, and underweight among children under 5 years and remarkable progress in optimizing iodine status among school-aged children were reported. Conversely, the burden of low birthweight and overweight/obesity exacerbated, coexisting with anemia, vitamin A deficiency, and vitamin D deficiency. Overall, fruit and vegetable consumption were inadequate. The consumption of soft drinks and salt on the other hand was higher than recommended. This review acknowledges the double burden of malnutrition in Jordan and recommends the prioritization and evaluation of interventions towards improving the population’s nutritional status and achieving nutrition targets.

## 1. Introduction

Malnutrition, the number one contributor to the burden of disease, remains a major global public health problem [1,2]. The Eastern Mediterranean Region (EMR) is no exception with many countries experiencing a double burden of malnutrition, involving undernutrition and micronutrient deficiencies coupled with elevated rates of overweight, obesity, and non-communicable diseases (NCDs) [3,4,5]. Despite some progress, key drivers of malnutrition have been shown to persist especially among infants, young children, adolescents, and women [3,5]. Throughout the different stages of the life-cycle, malnutrition has been shown to hinder individuals’ physiological, sensory, or mental health and wellbeing [6]. Undernutrition in children, especially in the early stages of life, is a risk factor for impaired physical and cognitive growth and a weakened immunity [6,7,8,9]. Additionally, childhood obesity is associated with short-term adverse health effects including psychological, physiological, and metabolic complications, as well as long-term consequences including an increased risk of premature death, disability, and NCDs later in life [10,11]. In women, malnutrition can increase the likelihood of poor fetal development, suboptimal birthweight, complications during pregnancy, maternal mortality, and NCDs [6,9,12]. Moreover, malnutrition hinders economic development, particularly through imposing elevated healthcare expenses and decreasing productivity [1].

Jordan, a middle-income country in the EMR [13], is witnessing an escalating pace of nutrition transition that may be associated with an increased burden of malnutrition and related NCDs [3,14]. Combating malnutrition in all its forms is challenging and requires the need to combine multifactorial interventions to secure the provision and accessibility to healthy and sustainable diets [4,5]. These interventions should be led by a comprehensive analysis of the nutrition status in Jordan, which is crucial for developing evidence-based country-specific policies and strategies, prioritizing action plans, and measuring progress. To the best of our knowledge, findings on the nutrition situation in Jordan are lacking. Therefore, the purpose of this paper is to examine the available evidence and secular trends pertinent to specific nutrition-related indicators including infant and young child feeding (IYCF) practices; low birthweight (LBW); stunting, wasting, and underweight among children under 5 years; micronutrient deficiencies including anemia, vitamin A, vitamin D, and iodine deficiencies; overweight/obesity; and food consumption patterns among different age groups; as well as available information on current national nutrition policies and strategies in Jordan. This review serves as a tool to better recognize critical data gaps and guide context-specific recommendations or improvements in policies and actions targeting the nutrition situation in Jordan. The suggested interventions, which have high political support and interest for United Nations agencies and donor communities, aim to address the double burden of malnutrition and achieve the recommendations of the International Conference on Nutrition (ICN)-2, global targets for nutrition, NCDs, and Sustainable Development Goals.

## 2. Methods

The literature search was conducted between 18 May and 25 October 2021 using the following electronic databases: PubMed, Scopus, Google Scholar, Research Gate, United Nations International Children’s Emergency Fund (UNICEF) IYCF databases [15], UNICEF malnutrition databases [16,17], World Health Organization (WHO) The Global Health Observatory [18], WHO Global School-based Student Health Survey (GSHS) [19], WHO STEPwise Approach to NCD Risk Factor Surveillance (STEPS) Country Reports [20], and governmental websites in Jordan. The search was restricted to nationally representative studies and to material published in the English language. Trends in the prevalence of various indicators were presented when national data were available at different time periods, and specific nutrition indicators were evaluated in comparison to the World Health Assembly (WHA) targets for 2025 [21].

For IYCF practices, based on the availability of data, eight of the indicators proposed by WHO and UNICEF were reviewed [22]. Breastfeeding indicators included: exclusive breastfeeding under 6 months, early initiation of breastfeeding within one hour of birth, ever breastfed (0–23 months), and continued breastfeeding (12–23 months). Complementary feeding indicators included: introduction of solid, semi-solid, or soft foods (6–8 months old); minimum acceptable diet (6–23 months); minimum dietary diversity (6–23 months); and minimum meal frequency (6–23 months). The percentage of formula-fed infants under 6 months of age was also reported. Prevalence of LBW was defined as the percentage of infants weighing < 2500 g at birth [23]. Stunting, wasting, and underweight among children under 5 years were defined as height-for-age z score < −2 standard deviations (SD), weight-for-height z score < −2 SD, and weight-for-age z score < −2 SD, respectively [24]. For the interpretation of the thresholds and corresponding labels pertinent to stunting and wasting, the criteria defined by WHO-UNICEF were used [25].

Anemia was based on hemoglobin (Hb) levels, defined as Hb < 11 g/dL for children under 5 years and for pregnant women and as Hb < 12 g/dL for non-pregnant WRA [26]. Anemia was categorized as mild, moderate, or severe based on the following Hb cut-offs: children under 5 years and pregnant women: 10–10.9 g/dL, 7–9.9 g/dL, and <7 g/dL respectively; non-pregnant WRA: 11–11.9 g/dL, 8–10.9 g/dL, and < 8 g/dL respectively [26].

Iron deficiency was based on serum ferritin (SF) levels, defined as SF < 12 µg/L for children under 5 years and as SF < 15 µg/L for pregnant women and non-pregnant WRA [27]. Iron deficiency anemia was based on Hb and SF levels, defined as: Hb < 11 g/dL and SF < 12 µg/L for children under 5 years; Hb < 11 g/dL and SF < 15 µg/L for pregnant women; and Hb < 12 g/dL and SF < 15 µg/L for non-pregnant WRA [26,27]. The interpretation of the prevalence of anemia, iron deficiency, or iron-deficiency anemia as public health problems was based on the following classifications: no public health problem, ≤4.9%; mild public health problem, 5–19.9%; moderate public health problem, 20–39.9%; severe public health problem, ≥40% [26,27,28].

Vitamin A deficiency among children under 5 years and WRA was assessed based on serum retinol concentrations defined at ≤0.70 μmol/L. The magnitude of vitamin A deficiency as a public health problem was categorized based on the following prevalence ranges: mild public health problem, 2–9%; moderate public health problem, 10–19%; severe public health problem, ≥20% [29].

Definitions of vitamin D deficiency and insufficiency vary in the literature [30,31,32]. In this review, vitamin D status was interpreted based on serum 25-hydroxyvitamin D (25(OH)D) concentrations using three common cutoffs (<12 ng/mL, <20 ng/mL, and <30 ng/mL) depending on the criteria published in the published articles [31,32]. Among children under 5 years and WRA, individuals with 25(OH)D levels < 20 ng/mL were considered to have vitamin D insufficiency, while those with 25(OH)D levels < 12 ng/mL were considered vitamin D deficient [31]. Among adults, 25(OH)D < 30 ng/mL was defined as low vitamin D status and that <20 ng/mL as vitamin D deficient [32].

Iodine status among school-aged children was assessed based on the median urinary iodine concentration (UIC), total goiter prevalence, and household consumption of iodized salt. Using median UIC, the following classifications were used for the interpretation of iodine status: insufficient iodine intake and iodine deficiency, <100 μg/L; adequate iodine intake and adequate iodine nutrition, 100–199 μg/L; above the required iodine intake and posing a slight risk of more than adequate intake, 200–299 μg/L; excessive iodine intake and risk of adverse health consequences, ≥300 μg/L [33]. Using total goiter prevalence, the severity of iodine deficiency was assessed according to the following criteria: none, ≤4.9%; mild, 5–19.9%; moderate, 20–29.9%; severe, ≥30% [34].

For overweight and obesity, this review assessed the prevalence based on the criteria used in the originally published resources. Among children under 5 years and adults, the WHO criteria were used as follows: children under 5 years: weight-for-height z score > 2 SD; adults: body mass index (BMI) ≥ 25 kg/m^2^ [10]. For school-age children and adolescents, the WHO criteria (BMI-for-age z score >1 SD) [10] or Center for Disease Control and Prevention’s growth charts (BMI-for-age ≥85th percentile) [35] were used. For the interpretation of the thresholds and corresponding labels pertinent to overweight/obesity, the criteria defined by WHO-UNICEF were used as follows: very low, <2.5%; low, 2.5% to <5%; medium, 5% to <10%; high, 10% to <15%, and very high, ≥15% [25].

For food consumption patterns, available national data pertinent to food groups were retrieved and evaluated. For nutrition-related national programs and policies implemented in Jordan, information was extracted from governmental websites, individual studies, and review papers available in the literature.

## 3. Results

### 3.1. Infant and Young Child Feeding Practices

#### 3.1.1. Breastfeeding Indicators

**Figure 1:**
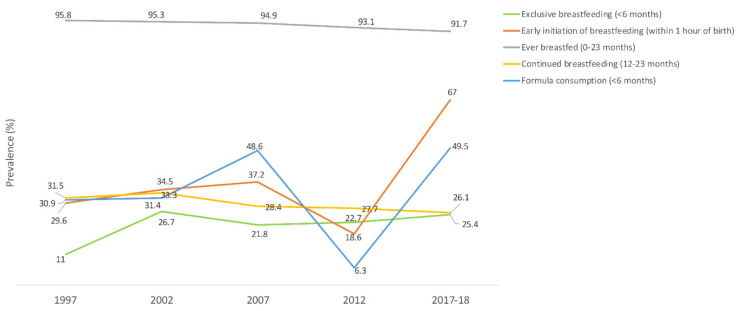
Trends in the prevalence (%) of breastfeeding indicators in Jordan, national surveys (1997–2017/18). Source: UNICEF, 2021; Neves et al., 2021 [15,37].

The prevalence of exclusive breastfeeding under 6 months of age was 25.4% in 2017‒2018 [36]. Rates decreased with the infant’s age, from 42.8% among children aged 0–1 month to 23.3% among those aged 2–3 months and 10.9% among those aged 4–5 months [36]. Two-thirds of infants (67%) were breastfed within the first hour of birth, and 91.7% of children were breastfed at some point between 0 and 23 months of age. As for continued breastfeeding, only 26.1% of 12–23 months old children were breastfed, of which 36% were at the age of 1 year and 15% at their second birthday [36]. The proportion of formula-fed infants below 6 months of age was almost 50% [37]. In terms of trend, the rate of exclusive breastfeeding under 6 months of age and that of early initiation of breastfeeding within 1 hour of birth fluctuated with an overall increase [15,37]. The proportion of ever breastfed children (0–23 months) and that of continued breastfeeding (12–23 months) decreased [15], while that of formula consumption (<6 months) increased over time [37]. More details are shown in Figure 1.

#### 3.1.2. Complementary Feeding Indicators

More than 8 out of 10 (83%) Jordanian children were introduced to solid, semisolid, or soft foods at 6–8 months of age in 2017–2018, as compared to a higher prevalence of 92% that was recorded in 2012 [15]. In terms of feeding practices, the minimum acceptable dietary standards were met by only 23% of children aged 6–23 months old, with 35% having an adequately diverse diet and 62% meeting the minimum meal frequency required for their age group [15,36]. When compared to earlier national data in 2012, the minimum acceptable diet, minimum diet diversity, and minimum meal frequency among 6–23 months old children showed a decreased proportion (33%, 39%, and 81%, respectively) [15]. Trends of complementary feeding indicators over longer periods were not derived due to the limited availability of data.

### 3.2. Low Birthweight

**Figure 2:**
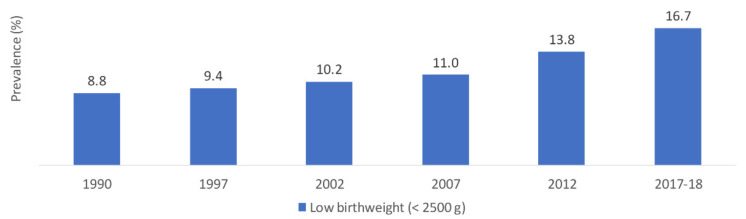
Trends in the prevalence (%) of low birthweight (<2500 g) in Jordan, national surveys (1990–2017). Source: DOS, 1992; DOS, 1998; DOS, 2003; DOS, 2008; DOS, 2013; DOS, 2019; Islam et al., 2020 [36,38,39,40,41,42,43].

National data collected over time reported an increasing trend in the prevalence of LBW, which almost doubled between 1990 and 2017–2018 (8.8% and 16.7%, respectively) (Figure 2) [36,38,39,40,41,42,43].

### 3.3. Stunting, Wasting and Underweight among Children under 5 Years

**Figure 3:**
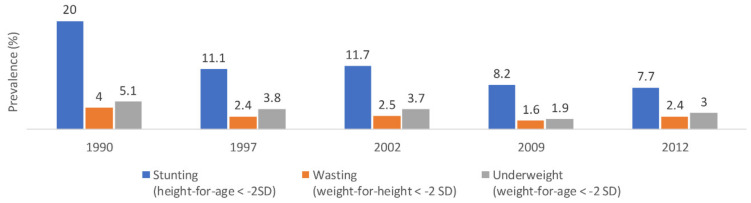
Trends in the prevalence (%) of stunting, wasting, and underweight among children under 5 years in Jordan, national surveys (1990–2012). Abbreviations: SD: standard deviation. Source: UNICEF, 2021 [16,17].

National data in 2012 reported that the rates of stunting, wasting, and underweight among children under 5 years were 7.7%, 2.4%, and 3%, respectively [39]. The prevalence of stunting was higher among males compared to females (9.2% and 6.1%, respectively) and among children aged 0–23 months old compared to 24–59 months (9.6% and 6.6%, respectively). The rates of wasting were comparable between males and females and between children aged 0–23 months and 24–59 months (2.5% and 2.4%, respectively). Underweight was more prevalent among males (3.3%) compared to females (2.7%) and among 0–23 months old (3.5%) compared to 24–59 months old (0.9%) [16,39]. Over time, the prevalence of stunting showed a decreasing trend from 20% in 1990 to 7.7% in 2012 (Figure 3) [16,39], with an average annual rate of change estimated at −2.8%. These findings were in line with modeled data reporting a decreasing trend in the prevalence of stunting [16,44]. As for wasting and underweight, the prevalence rates slightly fluctuated but showed an overall decreasing trend (4% and 5.1% in 1990 vs. 2.4% and 3% in 2012, respectively) (Figure 3) [16,39].

### 3.4. Micronutrient Deficiencies

#### 3.4.1. Anemia

##### Children under 5 Years

Based on the latest national Jordan Population and Family Health Survey (JPFHS) study in 2017–2018, the overall prevalence of anemia among children aged 6–59 months was reported at 32%. Most anemic children (21%) had mild anemia, whereas 11% and <1% had moderate and severe anemia, respectively [36]. When compared to earlier national studies, the prevalence of anemia was stable at 34% for seven years from 2002 to 2009, after which it slightly decreased to 32% in 2012 and 2017–2018 [36,45]. Among children aged 12–59 months, two national micronutrient surveys conducted in 2002 and 2010 measured the prevalence of anemia, iron deficiency, and iron deficiency anemia. Findings showed a significant decrease in the prevalence rates of iron deficiency (26.2% in 2002 and 13.7% in 2010) and iron deficiency anemia (10.1% in 2002 and 4.8% in 2010), and a non-significant decline in anemia (20.2% in 2002 and 17% in 2010) [46,47]. Modeled data showed that the prevalence of anemia among children aged 6–59 months old fluctuated, starting with a decreasing trend from 32.6% in 2000 to 30.4% in 2010 followed by an increase to reach 32.7% in 2019 [48].

##### Women of Reproductive Age

Findings from the latest national JPFHS survey in 2017–2018 showed that, among women aged 15–49 years, the overall prevalence of anemia was 43%. Mild and moderate anemia were found among 36% and 6% of women, respectively [26,36]. Compared to earlier JPFHS surveys, the prevalence of anemia among WRA showed an escalating trend starting from 26% in 2002 [36]. Moreover, among non-pregnant women ages 15–49 years, data from the national micronutrient survey indicated that the prevalence rates of anemia, iron deficiency, and iron deficiency anemia were 30.6%, 35.1%, and 19.8%, respectively [46].

#### 3.4.2. Vitamin A Deficiency

##### Children under 5 Years

Among children ages 12–59 months, national data showed an increasing trend in the prevalence of vitamin A deficiency from 15.1% in 2002 to 18.3% in 2010 [45,46,49].

##### Women of Reproductive Age

According to national data in 2010, the prevalence of vitamin A deficiency among non-pregnant women aged 15–49 years was 4.8%. The risk of deficiency was higher in women of younger age, who were unmarried, or living in rural areas [45,46]. National representative studies assessing vitamin A status among WRA in Jordan over time are lacking.

#### 3.4.3. Vitamin D Deficiency

##### Children under 5 Years

National findings in 2010 showed that 56.5% of children aged 12–59 months had vitamin D insufficiency (<20 ng/mL), and 19.8% had vitamin D deficiency (<12 ng/mL). Additionally, the prevalence of deficiency was significantly higher among females compared to males (25.9% and 14%, respectively) [50]. The scarcity of studies assessing the prevalence of vitamin D deficiency limited the assessment of trends over time.

##### Adults and Women of Reproductive Age

A nationally representative study conducted in 2017 showed that 89.7% of adults (>17 years old) had low vitamin D levels (<30 ng/mL), with higher rates reported in males (92.4%) compared to females (88.6%). As for vitamin D deficiency (<20 ng/mL), the prevalence was 71.2% and was higher among females compared to males (78.5% vs. 54%) [51]. Much lower estimates were recorded by a previous nationally representative study by Batieha et al. [52] where 29.4% of adults (≥19 years old) had low vitamin D levels (<30 ng/mL). Significantly higher prevalence rates were observed among females (37.3%) compared to males (5.1%) [52]. Moreover, El-Khateeb et al. [51] re-measured samples from Batieha et al. [52] using similar assays and indicated that 91% of the study population had low vitamin D levels (<30 ng/mL).

Among women aged 15–59 years, findings from El-Khateeb et al. (2019) showed the prevalence rates of having vitamin D levels < 30 ng/mL and <20 ng/mL were 89% and 80%, respectively [51]. Another nationally representative study conducted among non-pregnant WRA (15–49 years) in 2010 showed that 95.7% and 60% of participants had levels of vitamin D < 20 ng/mL and <12 ng/mL, respectively [53,54].

#### 3.4.4. Iodine Deficiency

A national iodine study conducted in 2010 among school-aged children aged 8–10 years old showed that the median UIC was 203 μg/L [46,55], a value much higher than previous national findings (154 μg/L in 2000 and 40 μg/L in 1993) [45,46,55,56]. Moreover, the prevalence of goiter showed a sharp decreasing trend, where rates dropped from 37.7% in 1993 and 33.5% in 2000 to 4.9% in 2010 [45,46,55,56]. Some disparities in the rates were reported among governorates such as Amman, Balqa, Jarash, and Ma’an where iodine deficiency persisted as a mild public health problem [45]. At the household level, the consumption of iodized salt showed an escalating trend from 88.3% in 2000 to 96.4% in 2010 [46].

### 3.5. Overweight and Obesity

#### 3.5.1. Children under 5 Years

**Figure 4:**
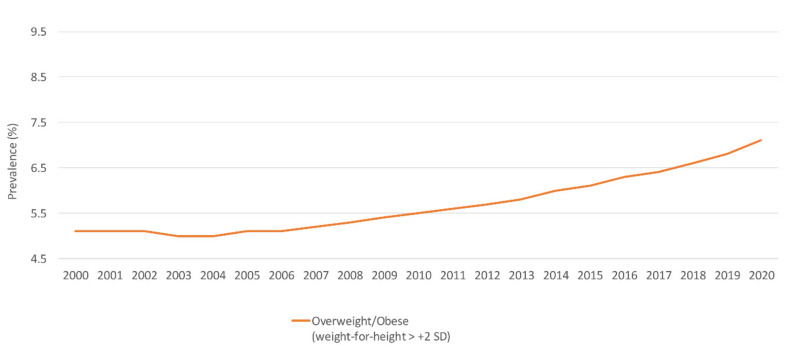
Trends in the prevalence (%) of overweight/obesity among children under 5 years in Jordan, modeled data (2000–2020). Abbreviations: SD: standard deviation. Source: WHO, 2021 [44].

According to national data in 2012, the prevalence of overweight and obesity was 4.7% among children under 5 years, with higher rates observed among males (5.6%) as compared to females (3.8%) [39]. National survey data reported a fluctuating trend in the prevalence of overweight and obesity over time [16,39]. Available modeled data suggested an escalating trend (Figure 4) [44].

#### 3.5.2. School-Aged Children and Adolescents

**Figure 5:**
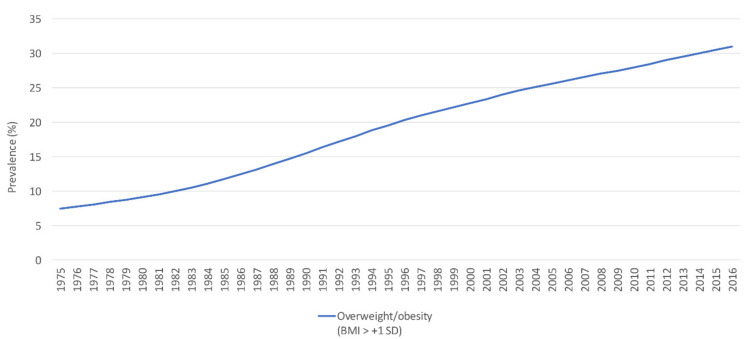
The trend in the prevalence (%) of overweight/obesity (BMI > + 1 SD) among school-aged children and adolescents (5–19 years old), modeled data (1975–2016). Abbreviations: BMI: body mass index; SD: standard deviation. Source: WHO, 2021 [60].

Among 6- to 17-year-old children and adolescents, national data in 2015–2016 indicated that the prevalence of overweight (BMI-for-age ≥ 85th–<95th percentile) was 17.3% and that of obesity (BMI-for-age ≥ 95th percentile) was 15.7%. Overweight was more common among females compared to males (18.9% and 15.3%, respectively), while obesity was more common among males than females (18.9% vs. 11.2%) [57]. Moreover, according to GSHS data collected in 2007 among students aged 13–15 years, 14.3% and 3.9% were categorized as having BMI-for-age ≥ 85th–<95th percentile and ≥95th percentile, respectively [58]. Slightly lower results were documented by GSHS data in 2004 (13.9% and 3.5%, respectively) [59]. According to modeled data, the prevalence of overweight and obesity (BMI > +1 SD) among 5- to 19-year-olds showed an increasing trend from 7.5% in 1975 to 31% in 2016 (Figure 5) [60].

#### 3.5.3. Adults and Women of Reproductive Age

**Figure 6:**
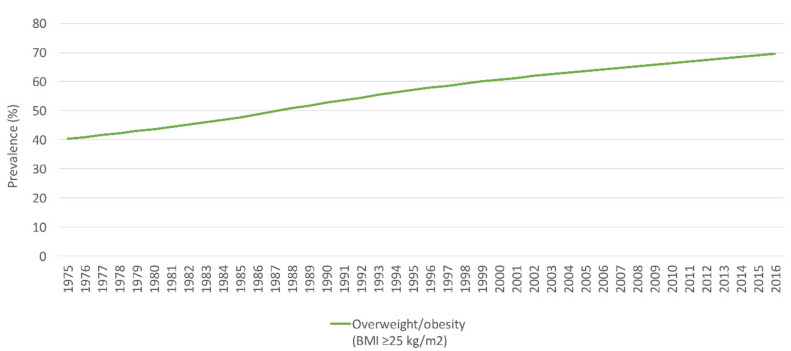
The trend in the prevalence (%) of overweight and obesity (BMI ≥ 25 kg/m^2^) among adults in Jordan, modeled data (1975–2016). Abbreviations: BMI: body mass index; kg: kilogram; m: meter. Source: WHO, 2021 [65].

Based on recent national data from the 2019 STEPS survey, the prevalence of overweight and obesity (BMI > 25 kg/m^2^) was 60.8%. The proportion of overweight (BMI 25–29.9 kg/m^2^) was 28.7% and was comparable between males (29%) and females (28.4%). The prevalence of obesity (BMI > 30 kg/m^2^) was 32.1% and was more common among females (40.4%) compared to males (24.2%) [61]. Findings from STEPS surveys over time showed that the prevalence of overweight and obesity among adults fluctuated, with an overall increase from 57% in 2004 to 60.8% in 2019 [61,62,63]. Moreover, modeled data estimated an increasing trend of overweight and obesity from 36.4% in 1975 to 66.5% in 2016 (Figure 6) [64].

Among women aged 18–44 years, the prevalence of overweight and obesity in 2019 reached 60%, of which 30% were overweight and 30% were obese [61]. Compared to earlier national surveys, the rates of overweight and obesity showed a fluctuating trend [36,39,40,61], with rates predicted to reach 75% in 2030 [66].

### 3.6. Food Consumption Patterns

#### 3.6.1. Children under 5 Years and Adolescents

Among children aged 6–23 months, national data showed an increasing trend in the proportions of children not consuming fruits and vegetables on a daily basis. The prevalence of not consuming any fruits or vegetables during the day preceding the survey almost doubled between 2007 and 2017–2018 (21.3% in 2007, 29.5% in 2012, and 41.1% in 2017-2018) [15]. Among adolescents aged 13–15 years, the comparison of national GSHS data (2004 vs. 2007) showed a reduction in the overall daily consumption of fruits and vegetables. Consuming fruits one or more times per day during the 30 days preceding the survey decreased from 73.9% to 69.2% in this age group, and that of vegetables declined from 80.4% to 77.5%, from 2004 to 2007, respectively. The overall consumption of five or more fruits and vegetables per day during the 30 days preceding the survey remained stable (24.7% in 2004 and 25.2 in 2007) [58,59]. Conversely, an increase in the consumption of fast food, three or more times during the seven days preceding the survey, was also recorded (11.4% in 2004 and 14.6% in 2007) [58,59]. Similar trends were observed when assessing the percentage of soft drinks’ consumption two or more times per day and that of milk or milk products three or more times per day during the 30 days preceding the survey (27.8% vs. 38.1% and 16.4% vs. 35.8%, in 2004 and 2007, respectively) [58,59].

#### 3.6.2. Adults and Women of Reproductive Age

According to the national STEPS survey conducted in 2019 among adults (18–69 years old), the average consumption of fruits was 1 serving a day for 3 days per week, and that of vegetables was 2 servings a day for 6 days per week. The average consumption of both fruits and vegetables was equivalent to 3 servings per day, and the prevalence of consuming <5 servings per day was 84.4%. Similar data were documented between males and females and among women aged 18–44 years [61]. Findings from a previous STEPS survey in 2007 showed that adults consumed an average of 2 servings per day of fruits for 4 days per week and an average of 3 servings per day of vegetables for 6 days per week, and 14.2% reported consuming <5 servings of fruits and vegetables per day [67].

In terms of salt intake, the 2019 national STEPS survey showed that the mean estimated amount consumed among adults (18–69 years old) was 11 g/day, with slightly higher levels reported among males compared to females (12.5 g/day vs. 9.6 g/day). Women aged 18–44 years consumed 9.7 g of salt per day. Furthermore, 30.6% of adults always or often added salt to their food, and a high proportion was recorded among women, particularly those aged 18–44 years (33.6%). The addition of salt when cooking or when preparing food at home was reported among 79.3% of adults, and similar proportions were documented between genders and among women aged 18–44 years. Processed foods of high salt content were always or often consumed by 33.4% of adults, particularly females aged 18–44 years (38.8%) [61].

### 3.7. National Nutrition Policies and Strategies in Jordan

Jordan is one of the few countries in the EMR which responded to the recommendations of the ICN-1 and accordingly developed a “National Plan of Action” in 1996 [68], which was then further reviewed and successfully implemented. Another plan of action was developed in 2006 and updated in 2010 by the Ministry of Health and WHO in coordination with all sectors. Despite the challenges faced, Jordan created an enabling environment for setting up successful national nutrition programs [68].

#### 3.7.1. The Baby Friendly Hospital Initiative (BFHI) and the International Code of Marketing of Breast-Milk Substitutes

The baby friendly hospital initiative (BFHI) was launched in 1991 by UNICEF and WHO as a means of protecting, promoting, and supporting breastfeeding [69]. The revised initiatives in 2018 required the implementation of the International Code of Marketing of breast-milk substitutes (The Code) [70]. In Jordan, although the Code is adequately promoted to the general public and in health facilities, it has been shown to lack proper implementation and lacks adequate monitoring and evaluation [71]. Only three hospitals were designated for the BFHI, without proper monitoring and follow-up [72].

#### 3.7.2. Food Fortification and Micronutrient Supplementation

Since April 2006, Jordan implemented a mandatory national wheat flour fortification program, which initially comprised iron and folic acid, followed by zinc, vitamins A, B1, B2, B3, B6, and B12, after which vitamin D was added in 2010. The monitoring system included internal methods undertaken by the millers and external monitoring by the government, where non-compliant mills were subject to fines [54]. The program witnessed financial challenges related to funding the premix and the cost of the subsidized bread for refugees, in addition to the need of training employees in the mills across the country. Priority actions included assessing the efficiency of the program and strengthening its enforcement through enhanced capacity building, finances, and reporting systems. Moreover, in response to alarming suboptimal iodine levels in 1993, a mandatory universal salt iodization program was introduced in 1995 and has been efficiently progressing to date [45].

Additionally, as means of decreasing vitamin A deficiency, a vitamin A supplementation program was initiated in 2005. This program provided one shot of 100,000 IU of vitamin A at the time of measles vaccination at 10 months of age, followed by a second shot of 200,000 IU when giving the measles, mumps, and rubella vaccination (MMR) at 18 months of age. In 2008, the national higher committee of nutrition in Jordan modified this process by maintaining only the first dose of 100,000 IU. Then in 2012, in response to the increased prevalence of vitamin A deficiency, the national higher committee of nutrition retained the second dose of 200,000 IU vitamin A and requested to continuously monitor and evaluate the distribution of vitamin A capsules and documents provided in the national vaccines card [28,72].

#### 3.7.3. Multisectoral Coordination to Tackle Obesity

As a means of tackling nutrition-related challenges in Jordan, the National Framework of Action on Obesity Prevention (2018–2023) was founded in 2019 as a multisectoral approach. In addition to enhancing the quality and quantity of dietary fat and oils, the committee’s agenda included optimizing salt and sugar content in the diet, decreasing sedentary behavior, and enforcing marketing policies on food commodities and their labeling and breast milk substitutes. The committee managed to reduce the proportion of salt in bread, optimize the fat content of dairy products and food served in public institutions especially by eliminating industrially-produced trans-fat, and enhance the quality of food served in youth camps. Educational material including country-specific food consumption tables, food-based dietary guidelines, and dietary recommendations for managing NCDs were also provided [73].

## 4. Discussion

This review highlighted the overall suboptimal status of breastfeeding and complementary feeding indicators among infants and young children. Although the majority of children in Jordan were breastfed at some point in their young lives and early initiation of breastfeeding showed a two-fold increasing trend over time, the prevalence of exclusive breastfeeding during the first six months of life was only 26% [15,36]. This rate of exclusive breastfeeding is much lower than the global average (44%), lower than the estimate reported for the EMR (34%) [15], while also being below the WHA target of 50% by 2025 [21,37]. Of more concern is the more or less stable trend of exclusive breastfeeding that was observed over a period of 15 years since 2002, coupled with the decreased rates of continued breastfeeding throughout the child’s second year and the increased proportion of formula-fed infants [15,36,37,74]. This poor adherence to the WHO infant feeding recommendations may have negative consequences on the burden of diseases in Jordan. Evidence has shown that adequate early infant nutrition may be a crucial factor in improving cognitive development and physical growth, enhancing immunity, decreasing the risk of childhood obesity, and preventing NCDs [75,76]. The suboptimal status of the aforementioned breastfeeding indicators may be attributed to poor implementation of the BFHI and the Code [37,72,77]. Other potential challenges could be the lack of sufficient breastfeeding knowledge among healthcare providers and their key role in encouraging lactating mothers [72]. Inadequate breastfeeding practices and premature weaning may also be due to the lack of designated maternity facilities such as lactation rooms in shopping malls, airports, and the workplace, lack of community or home support to the breastfeeding mother, high rates of prelacteal feeding, and the growth of infant formula milk companies in the market [37,72,77,78,79,80]. In terms of complementary feeding, although most infants were introduced to solid, semi-solid, or soft foods as per the WHO recommendations [15], less than half of those aged 6–23 months received a minimum acceptable diet and minimum diet diversity. A decreasing trend, rather than improvement, was recorded in all the core complementary feeding indicators hence requiring interventions [15,81]. Studies have shown that breastfeeding may be associated with higher diet diversity and lower consumption of sugar, fat, and salt [81,82]. One hypothesis is that caregivers who follow recommendations pertinent to breastfeeding are more likely to adhere to other guidelines such as the provision of healthier foods and behaviors [82]. Another explanation is that breast milk offers infants early exposure to a variety of flavors, which has been shown to enhance their food acceptance and food choices [82,83]. Moreover, the education level and knowledge about feeding practices of mothers in Jordan were shown to be significant determining factors to the adequate diet of children aged 6–23 months [81], which was in line with other findings in the literature [84,85,86,87]. Other crucial elements affecting complementary feeding practices include low socio-economic status, food insecurity, as well as traditional cultural practices such as the provision of sugar-sweetened water for treating jaundice in children in certain rural areas [81,88,89,90,91,92].

The findings of this review showed that LBW is a public health challenge in Jordan, given its continuously increasing prevalence rates, indicating poor alignment with the WHA target for 2025 [21,38]. Moreover, the estimated rate of LBW in Jordan exceeded the 2015 global (14.6%) and regional average estimates, except for Southern Asia (26.4%) [93,94]. The status of LBW is of concern given its potential consequences on impairing growth, cognitive development, and immunity among other adverse health effects [3]. Potential factors triggering the increasing trend in LBW may include suboptimal maternal health and nutrition, illness, short interpregnancy intervals, and epidemiological transition [3,38].

Based on the WHO anthropometric cutoffs of stunting, wasting, and underweight [95], this review documented a low burden of undernutrition among children under 5 years in Jordan. The prevalence of stunting was low and showed an overall decreasing trend, despite its stagnation over a period of five years between 1997 and 2002 [16,25,96]. This temporary lack of change may have been attributed to some population groups’ inaccessibility to nutrient-dense foods or lack of knowledge pertinent to care and hygiene practices [96]. Moreover, the annual rate of change in the prevalence of stunting was estimated at −2.8%, a value below the WHA target for 2025 (−3.9%) [21,25]. The prevalence of stunting in Jordan was lower than the global average of 22% and below the rates reported from all WHO regional categories except Europe (5.7%) [16,25]. As for wasting, the prevalence rate was very low and met the WHA target for 2025 (<5%) [21,25]. The rate of wasting among children under 5 years in Jordan was lower than the worldwide average (6.7%) and rates reported from South-East Asia (14.5%), EMR (7.4%), and Africa (5.8%), although it was comparable to the Western Specific Region (2.1%) and exceeded the Americas (0.7%) [16,25]. Moreover, an overall decreasing trend in the prevalence of underweight was observed [16]. The rate in Jordan was found to be below the global average of underweight (12.6%) and those reported from South-East Asia (24.8%), Africa (15.7%), and EMR (12.1%), but slightly exceeded Regions of the Western Specific (2.3%) and Americas (1.7%) [97]. The overall decreasing trend in stunting, wasting, and underweight may have been associated with several factors including the improvement of maternal education levels, the gradual increase in the number of women’s and children’s health centers, the increased proportion of women receiving antenatal care, and the improved vaccination coverage reported in Jordan [39,98,99,100,101,102].

This review underlined the persistence of anemia as a challenge among children under 5 years and among WRA in Jordan. In children aged 6–59 months old, the prevalence in 2017–2018 was classified as a moderate public health problem [26]. Among children aged 12–59 months, the rates of anemia and iron deficiency in 2010 were categorized as mild public health concerns, while that of iron deficiency anemia was considered normal [26,27,28,46]. Over time, the rates of anemia did not show significant progress, whereas a decreasing trend in the prevalence of iron deficiency was observed, which may be attributed to the implementation of the national wheat flour fortification with multiple micronutrients including iron [47]. However, to establish a more accurate interpretation of the role of the fortification program on iron status, additional data on the actual dietary intake is needed [47]. Moreover, given that the prevalence of iron deficiency anemia was substantially less (two to three times lower) than that of anemia [28,46], there is a need to further investigate other potential causes of anemia (in addition to iron deficiency) to implement appropriate interventions [28]. For instance, evidence reported a high prevalence of diarrhea among this population group, indicating the need to develop alternative processes, such as control of infectious diseases, to help decrease anemia [45]. As for WRA, the prevalence of anemia in 2017–2018 was classified as a severe public health problem [26]. Of more concern is the escalating prevalence over time [36,103], indicating a non-alignment with the WHA target for 2025 [21]. Among non-pregnant WRA, rates of anemia and iron deficiency in 2010 were categorized as public health concerns of moderate severity and that of iron deficiency anemia as a mild public health problem [26,28,46]. Potential factors responsible for anemia among WRA could be nutritional deficiencies, including iron deficiency as a major determinant, vitamin B12 and folate deficiencies [45,46]. This may be attributed to the decreased access to nutrient-dense and iron-rich foods caused by the significant increases in the food price index following the economic crises which began in 2008 [47,103,104]. Moreover, the persistence of iron deficiency anemia among non-pregnant women may be caused by the consumption of antinutritional compounds, the regular ingestion of tea with food which may decrease the bioavailability of iron, and the consumption of food low in iron [45,105,106]. Studies in the literature have presented other risk factors associated with iron deficiency anemia including heavy menstrual blood loss, multiple children, and the use of intrauterine devices as a contraceptive method [103,107].

As for the status of vitamin A, the deficiency was classified as a moderate public health problem among children under 5 years [29,45,108] and as a mild public health problem among WRA [29,45,108]. These findings may be explained by the possible suboptimal consumption of vitamin A-rich foods [45]. For instance, findings from JPFHS surveys showed that the percentage of children who consumed foods rich in vitamin A in the 24 h preceding the interview decreased from 83.5% in 2007 to 67.8% in 2012 and 67.2% in 2017-2018 [36,39,40]. Additional data on the actual quantity of vitamin A intake and the factors associated with the reported decreased consumption are needed.

For vitamin D status, among children under 5 years, moderately high prevalence rates of vitamin D deficiency (<12 ng/mL) and insufficiency (<20 ng/mL) were reported, particularly among females [45,50]. The gender difference in serum vitamin D levels may be due to females having less outdoor activities as compared to males hence synthesizing less vitamin D in the skin [109,110]. Among adults, findings in the literature reported wide discrepancies in the prevalence of vitamin D deficiency which could be explained by the studies’ use of different assays for the measurements of 25-hydroxyvitamin D [51]. Overall, the prevalence of low vitamin D levels among adults and WRA in Jordan was shown to be very high and among the highest in the EMR [54], with more severe deficiencies observed among females [51]. The gender disproportionality of vitamin D status may be attributed to cultural practices, such as the dressing style [50,52].

Remarkable progress has been achieved in optimizing the iodine status in Jordan, although some attention needs to be given to the few governorates where iodine deficiency persisted as a mild public health problem. The implementation of the iodine deficiency disorder program has been shown to efficiently contribute to the enhancement of the iodine status. This was reflected in the considerable increase in median UIC (+163 µg/L from 1993 to 2010) and the decline in the prevalence of goiter below the threshold of 5%, in parallel to the increased household consumption of iodized salt [45,46].

This review highlighted an alarming increase in the rates of overweight and obesity among the various age groups in Jordan. Among children under 5 years, medium prevalence (between 5% to less than 10%) of overweight and obesity was reported [25], and its escalating trend indicated a lack of progress in achieving the WHA target for 2025 [21]. Moreover, rates exceeded the global average of 5.7% and those of Africa (4.2%) and South-East Asia (3.3%) but were close to the average rates of the Americas (8%), Europe (7.9%), EMR (7.7%), and Western Pacific (7.5%) [16,25]. Among school-aged children and adolescents, findings showed an emerging public health concern whereby the proportion of overweight or obesity almost quadrupled over the past four decades, reaching 31% in 2016 [61]. This prevalence of overweight and obesity exceeded that reported globally by the WHO (25.2%) and in the EMR (28.7%), while being more than double the estimates reported in Africa (14.2%) and South-East Asia (11.9%), yet lower than rates reported in America (48%), Europe (34.8%) and Western Pacific (34.7%) [10,111,112,113]. Among adults, the elevated prevalence of overweight and obesity exceeded that reported globally by the WHO (52%). When compared to regional data, rates were higher than those of Western Pacific (39.4%), Africa (38.3%), and South-East Asia (26.2%), similar to the EMR (67.3%), but lower than America (92%) and Europe (85.6%) [111,112,113]. The escalating rates of overweight and obesity may be attributed to the country’s nutrition transition and lifestyle changes towards a westernized diet characterized by higher consumption of energy, sugar-sweetened beverages, and processed foods, coupled with decreased intakes of fruits and vegetables and adherence to more sedentary behaviors [114,115,116,117,118]. Moreover, the higher prevalence of overweight and obesity observed among women could be explained by the cultural constraints in lifestyle choices such as the increased barriers to physical activity as compared to men [119,120]. For instance, women in the EMR are expected to adhere to traditional responsibilities in societies including childcare and household chores which may limit their availability to engage in physical activity [121]. This escalating burden of overweight and obesity in Jordan may cause serious public health implications, considering their associations with NCDs and mortality [122,123,124].

This review documented a low intake of fruits and vegetables among different age groups in Jordan and a decreasing trend in the proportions of individuals achieving the WHO daily recommendations of ≥5 servings of fruits and vegetables per day [125]. Of more concern, these findings were coupled with other unfavorable food consumption patterns such as the increased intake of soft drinks among adolescents [58,59]. Moreover, despite the increased consumption of milk and dairy products over time, less than half of adolescents reached the recommended intake of three servings per day [126]. Comparable findings were reported by different cross-sectional studies in Jordan, which also documented high consumption of sweetened beverages, sweets and chocolate, cakes and cookies, French fries, and potato chips [127,128,129]. Among adults and WRA, high consumption of salt was reported where the mean intake levels reached double the WHO recommendation of less than 5 g/day [126]. These faulty food consumption patterns may reflect the ongoing nutrition transition towards a more westernized diet, which has been suggested to contribute to the escalating burden of obesity accompanied by micronutrient deficiencies, as well as increase the risk of NCDs during adolescence and into adulthood [3,129,130,131].

Although this review has provided valuable insight into the nutritional situation and food consumption patterns in Jordan, its findings should be considered in view of the following limitations. When assessing the nutrition-related indicators, interpretation was limited by the scarcity of recent and nationally representative data. This may have restricted the interpretation of additional vitamins and minerals, although scarce, and other population groups such as pregnant and lactating women and older adults. Challenges and gaps in the analysis of food consumption patterns were identified, particularly the lack of nationally representative individual-level dietary intake surveys. The paucity of data may be attributed to limited research funding, lack of coordination between different stakeholders, and unavailability of food composition tables.

## 5. Recommendations

The low rates of exclusive breastfeeding and continued breastfeeding highlight the need to strengthen national regulatory systems to effectively monitor and enforce the Code and BFHI-related indicators [72]. Priority interventions should also aim at reinforcing the skills of health professionals through capacity building to effectively implement those policies, as well as integrating educational materials into the academic medical curricula [72,132]. It is also important to incorporate community initiatives to address the challenges faced by breastfeeding mothers after their discharge from the hospital and throughout their second year postpartum [72]. As means of enhancing complementary feeding practices, it is crucial to design culture-specific nutrition awareness campaigns and education programs targeting all caregivers including fathers and grandparents, not just mothers, with emphasis on the less educated [81,84,133]. Moreover, the living conditions, food availability, and food security status among vulnerable populations and individuals residing in remote areas need to be improved to help enhance the diets of infants and young children [81]. The escalating prevalence of LBW highlights the need for governmental interventions and attention from national child health systems, in parallel to further investigating the underlying causes [3,38]. To help improve LBW rates, special attention needs to be given to maternal health and nutrition such as tackling anemia in a multi-level intervention rather than focusing on iron alone.

Additionally, the persistent suboptimal status of some micronutrients including iron, vitamin A and vitamin D calls for the need to evaluate their relevant national policies and strategies. For more accurate interpretation, it is recommended to conduct research studies assessing individual micronutrient intakes and supplementation. It is also essential to develop evidence-based awareness and education workshops targeting the underlying causes of micronutrient deficiencies while emphasizing diet diversification as a strategy. In terms of iodine status, although significant progress has been reported, the median UIC (203 μg/L) in 2010 was classified at the lower limit of the ‘above requirement’ category based on WHO/International Council for Control of Iodine Deficiency Disorders criteria [33], highlighting the need to carefully monitor the salt iodization program in Jordan [45,46]. To combat the escalating rates of overweight and obesity and the suboptimal food consumption patterns across the different age groups in Jordan, multidisciplinary evidence-based approaches need to be adopted. These may include awareness of optimal dietary patterns and lifestyle behaviors, in addition to policies targeting food systems to produce healthier foods including food reformulation laws and labeling regulations [134]. Another priority food system approach is to reduce the cost of nutritious foods for rights holders and to render healthy diets more affordable while ensuring reasonable prices for the producer. This would help prevent the double burden of malnutrition, lower the cost of healthcare-associated with unhealthy diets, reduce food waste, and save costs. In Jordan, shifting to sustainable and healthy consumption patterns requires developing procedures pertinent to the following three aspects: motivating and empowering consumers to make informed, healthy, safe, and sustainable alternatives; improving the availability, accessibility, and affordability of healthy safe and sustainable foods; and minimizing food waste originating from food service, retail, or household. Moreover, the WHO recommended the need for Jordan to endorse and preserve six food system actions which have been characterized as “game changers”, including fiscal policies for healthy and sustainable diets; public food procurement and service policies for a healthy diet sustainably produced; regulation of marketing of foods and non-alcoholic beverages including breastmilk substitutes; food product reformulation; front-of-pack labeling, and food fortification [135]. There is also a need to strengthen the implementation of the objectives of the National Framework of Action on Obesity Prevention [73] and set up an efficient monitoring and evaluation system. Moreover, the scarcity of data in the literature highlights the need for more recent nationally representative surveys, particularly individual-level dietary intake studies.

## Data Availability

Not applicable.
